# Training Medical Residents to Address Social Determinants of Health for Better Health Outcomes

**DOI:** 10.1093/geroni/igaa057.830

**Published:** 2020-12-16

**Authors:** Angela Zell, Joan Ilardo, Adesuwa Olomu, Cristian Meghea, Supratik Rayamajhi

**Affiliations:** Michigan State University, East Lansing, Michigan, United States

## Abstract

Gaps exist in training medical residents to assess social determinants of health (SDOH) related to chronic conditions. To address the need for better screening, we partnered with two Internal Medicine (IM) residency programs based in Lansing and Flint (Michigan) to pilot the Caring for Patients with Chronic Conditions (CPCC) project. IM residencies train internists with expertise in diagnosis, treating chronic conditions, promoting health through wellness education, and preventing and managing diseases. CPCC incorporated information during didactic sessions that residents could apply during their clinical activities that can influence their current and future clinical practice patterns. Presentations and panels from local community organizations on specific topics were incorporated into the curriculum that address needs of patients age 50 and older. To build on this education, the residents adapted the Office- Guidelines Applied in Practice (Office-GAP) checklist to identify SDOH affecting a patient’s ability to managed chronic conditions. Using this tool: 1) involves resident training; 2) provides a decision support checklist; 3) influences patient activation; and 4) increases provider and patient communication through shared decision making. The checklist includes questions for patient response pertaining to SDOH that prevents them from managing their chronic conditions in addition to the level of action the patient is willing to do. Areas identified are discussed between patient and resident increasing patient activation. Referrals to community-based resources to identified SDOH needs are guided by the clinic’s care manager. The Office-GAP tool is administered during three subsequent visits to ensure that patients actually accessed the community resources.

